# Stellungnahme zum Einsatz von topisch-inhalativem Budesonid bei COVID-19-Infektion: Positionspapier des Ärzteverbands Deutscher Allergologen (AeDA) und der Deutschen Gesellschaft für Hals-Nasen-Ohren-Heilkunde, Kopf- und Hals-Chirurgie (DGHNO-KHC)

**DOI:** 10.1007/s00106-021-01084-3

**Published:** 2021-06-17

**Authors:** Ludger Klimek, Roland Buhl, Thomas Deitmer, Stefan Plontke, Wolfgang Wehrmann, Hans Merk, Johannes Ring, Sven Becker, Ärzteverband Deutscher Allergologen (AeDA), Sven Becker, Ludger Klimek, Hans Merk, Johannes Ring, Wolfgang Wehrmann, Deutsche Gesellschaft für Hals-Nasen-Ohren-Heilkunde, Kopf- und Hals-Chirurgie (DGHNO-KHC), Thomas Deitmer, Stefan Plontke

**Affiliations:** 1 Zentrum für Rhinologie und Allergologie Wiesbaden, Wiesbaden, Deutschland; 2 Schwerpunkt Pneumologie, III. Medizinische Klinik und Poliklinik, Universitätsmedizin Mainz, Mainz, Deutschland; 3 Deutsche Gesellschaft für Hals-Nasen-Ohren-Heilkunde, Kopf- und Hals-Chirurgie, Bonn, Deutschland; 4 Klinik und Poliklinik für Hals-Nasen-Ohren-Heilkunde, Kopf- und Hals-Chirurgie, Martin-Luther-Universität Halle-Wittenberg, Halle, Deutschland; 5 Dermatologische Gemeinschaftspraxis Wehrmann, Münster, Deutschland; 6 Abteilung Dermatologie & Allergologie, RWTH Aachen, Aachen, Deutschland; 7 Haut- und Laserzentrum an der Oper, München, Deutschland; 8 Klinik für Hals-, Nasen- und Ohrenheilkunde, Kopf- und Halschirurgie, Eberhard Karls Universität Tübingen, Elfriede-Aulhorn-Str. 5, 72076 Tübingen, Deutschland

Glukokortikosteroide (GKS) gelten als Standardtherapie entzündlicher Erkrankungen der Atemwegsschleimhäute, wie allergische Rhinitis (AR), chronische Rhinosinusitis (CRS) oder Asthma bronchiale (A) [1, 2]. CRS, AR und A gehören zu den häufigsten entzündlichen Erkrankungen überhaupt, und mit der Chronifizierung sind häufig Epithelschädigungen und Gewebsdestruktionen verbunden, die Virusinfektionen Vorschub leisten können [1, 2].

Ein Asthma bronchiale ist eine wichtige Komorbidität von AR und CRS. Verschlechterungen in der Kontrolle von AR und CRS können Asthma-Exazerbationen begünstigen [3–7].

Nachdem in der aktuellen Pandemie durch SARS-CoV‑2 („severe acute respiratory syndrome coronavirus type 2“) zunächst Hinweise publik wurden, dass „Kortison-Präparate“ das Risiko erhöhen, an COVID-19 („coronavirus disease 2019“) zu erkranken, bzw. einen schwereren Verlauf der Erkrankung hervorrufen könnten und hierdurch zahlreiche Patienten mit AR, CRS und Asthma massiv verunsichert wurden, wiesen Positionspapiere der deutschen und europäischen Gesellschaften sehr frühzeitig in der Pandemie auf die Notwendigkeit einer Fortführung der Therapie mit topischen GKS hin [1, 2]. Demnach sind topische GKS als nasale (nGKS) und inhalative (ICS) Form effektiv in der Behandlung von Schleimhautentzündungen der oberen und unteren Atemwege und stellen die Standardtherapie dieser Erkrankungen dar [8–11].

Es existieren keinerlei Hinweise, dass eine Anwendung von nGKS und ICS ein erhöhtes Risiko für eine SARS-CoV-2-Infektion oder einen schwereren Verlauf einer COVID-19-Erkrankung auslösen. Erwachsene und Kinder mit AR, CRS und A sollten daher ihre verordneten nGKS und ICS konsequent und regelmäßig in der individuell verordneten Dosis einnehmen und nicht ohne Rücksprache mit dem behandelnden Arzt ändern oder gar beenden [1, 2]. Hierbei wiesen wir auch darauf hin, dass eine gute antientzündliche Kontrolle der oberen und unteren Atemwege durch topische GKS nach aktuellem Stand des medizinischen Wissens ein guter Schutz vor durch Viren ausgelöste Exazerbationen für diese Patienten darstellt [1, 2]. Aus heutiger Sicht gibt es genügend Daten, dass Patienten mit chronisch-entzündlichen Atemwegserkrankungen im Rahmen der COVID-19-Pandemie eine leitliniengerechte pharmakologische Behandlung erhalten sollten, die, falls erforderlich, nGKS, ICS und Biologika-Therapien einschließt [12, 13]. Zu den o. g. topischen Glukokortikosteroiden gehört auch Budesonid.

## Studie zur Anwendung von Budesonid bei COVID-19

In der letzten Ausgabe der Zeitschrift *THE LANCET Respiratory Medicine* veröffentlichten Ramakrishnan et al. eine hypothesengenerierende Studie über den Einsatz von inhalativem Budesonid im Vergleich zur „Standardtherapie“ bei Patienten mit frühem COVID-19 [14]. Die Autoren führten eine offene, randomisierte kontrollierte Parallelgruppenstudie der Phase II durch, um den Einsatz von inhaliertem Budesonid (1600 µg/Tag) mit der Standardtherapie bei Patienten mit in den vergangenen 7 Tagen festgestellter, symptomatischer COVID-19-Erkrankung zu vergleichen [14]. Die Autoren kommen zur Schlussfolgerung, dass diese Behandlungsform die erste kostengünstige und leicht zugängliche therapeutische Intervention bei COVID-19 im Frühstadium sein könnte. Sie diskutieren, dass ihre Daten auch eine potenziell wirksame Behandlung zur Verhinderung langfristiger und anhaltender COVID-19-Symptome darstellen könnte.

## Bewertung der Studiendaten

Die oben dargestellten Aussagen [14] werden unseres Erachtens durch die präsentierten Daten nicht unterstützt. Es handelte sich um eine offene Studie, bei der Patienten und Ärzte über die Art der Therapie informiert waren. Placeboeffekte von ICS bei Asthma bronchiale werden mit 21–46 % beschrieben, insbesondere wenn subjektive Ergebnisparameter als Evaluationskriterien („outcome parameter“) verwendet werden [15].

Die in dieser Studie festgestellten Effekte, einschließlich des primären Endpunkts (COVID-19-bedingte ambulante Vorstellung oder Hospitalisierung) und sekundärer Endpunkte (wie die Zeit bis zur von den Patienten subjektiv empfundenen klinischen Besserung) könnten durch die subjektive Wahrnehmung der betroffenen Patienten und ihrer behandelnden Ärzte beeinflusst worden sein [16].

Objektive Messwerte wie die Sauerstoffsättigung im Blut, die Körpertemperatur, spirometrische Befunde und die quantitative Viruslast mit SARS-CoV‑2 wurden als weitere sekundäre Endpunkte in der Studie verwendet, unterschieden sich in ihren Ergebnissen jedoch nicht zwischen den Gruppen. Die Studie war klein und umfasste nur 146 Teilnehmer – 73 wurden in die „Standardtherapie“ und 73 in die Budesonid-Gruppe randomisiert.

Ramakrishnan et al. stellen die Hypothese auf, dass die frühzeitige Verabreichung von inhalativem Budesonid die Wahrscheinlichkeit reduziert, dass eine dringende medizinische Versorgung benötigt wird, und die Zeit bis zur Genesung bei einem frühzeitig behandelten COVID-19 verkürzt [14]. In Anbetracht der Evidenz aus dieser und anderen Studien bleibt diese Interpretation der Daten spekulativ.

Der primäre Endpunkt wurde bei 11 (15 %) der Kontrollpatienten und 2 (3 %) der Budesonid-Patienten erreicht (*p* = 0,009). Die Zeit bis zur klinischen Besserung war bei den mit Budesonid behandelten Patienten um einen Tag kürzer (7 vs. 8 Tage, *p* = 0,007). Bei 2 % der Budesonid-Patienten trat in den 14 Tagen nach Studienbeginn Fieber auf, im Vergleich zu 8 % der Kontrollpatienten (*p* = 0,051). Unerwünschte Wirkungen traten bei 5 Patienten (= 7 %) in der Budesonid-Gruppe auf.

Hierzu passen die Zwischenergebnisse einer noch laufenden größeren Phase-III-Studie an 2617 Patienten, die ebenfalls eine inhalative Budesonid-Therapie bei akuter SARS-CoV-2-Infektion prüft [17]. Hier zeigte sich eine um 3 Tage kürzere Zeit bis zur selbstberichteten klinischen Besserung der Symptome, der geringe Effekt auf COVID-19-bedingte Hospitalisierungen oder Todesfälle (Budesonid: 59/692 [8,5 %] vs. 100/968 [10,3 %] Patienten) war allerdings nicht statistisch signifikant.

## Diskussion

Die deutschen Gesellschaften AeDA (Ärzteverband Deutscher Allergologen), DGP (Deutsche Gesellschaft für Pneumologie und Beatmungsmedizin), DGAKI (Deutsche Gesellschaft für Allergologie und klinische Immunologie), DGHNO-KHC (Deutsche Gesellschaft für Hals-Nasen-Ohren-Heilkunde, Kopf- und Hals-Chirurgie) gemeinsam mit internationalen Organisationen wie ARIA (Allergic Rhinitis and its Impact on Asthma Initiative), EAACI (European Academy of Allergy and Clinical Immunology) und GAL^2^EN (Global Allergy and Asthma European Network) betonten die Notwendigkeit der Fortführung und des konsequenten Einsatzes von ICS bei erkrankten Patienten bereits zu Beginn der Pandemie [12, 13]. Ähnliche Empfehlungen wurden auch für weitere allergische Erkrankungen während der Pandemie erstellt [18–20].

Die vorliegenden Studienergebnisse zur inhalativen Budesonid-Therapie sprechen dafür, dass bei Beginn einer inhalativen Budesonid-Therapie in hoher Dosis innerhalb weniger Tage nach Beginn einer COVID-19-Erkrankung eine etwas kürzere Symptomdauer erzielt werden kann. Ein höhergradiger klinischer Effekt lässt sich auf Basis der aktuellen Studienlage nicht belegen.

Eine vorsichtige Interpretation dieser Daten ist umso mehr angebracht, als eine aktualisierte Zwischenanalyse von Daten einer größeren Phase-III-Studie, die 2617 Personen mit Risikofaktoren für ungünstige Verläufe von COVID-19 einschloss, nicht so günstige Ergebnisse zeigte [17] – inhaliertes Budesonid verkürzte die Zeit bis zur selbstberichteten Genesung im Median um 3 Tage, erreichte aber nicht den primären Ergebnisparameter (vordefinierter Überlegenheitsschwellenwert für die Wahrscheinlichkeit, dass die Zahlen der COVID-19-Hospitalisierungen/-Todesfälle in der Budesonid-Gruppe im Vergleich zur Standardtherapie niedriger waren): Budesonid: 59/692 (8,5 %) vs. Standardtherapie: 100/968 (10,3 %) [17].

Neuere Daten geben des Weiteren Hinweise darauf, dass Patienten mit unterschiedlichen Asthma-Endotypen (Typ-2-Asthma vs. Nicht-Typ-2-Asthma) ein unterschiedliches Risikoprofil in Bezug auf die SARS-CoV-2-Infektion, die Entwicklung von COVID-19 und das Fortschreiten zu schweren COVID-19-Erkrankungen aufweisen [21].

Effektive Maßnahmen zur Eindämmung der Pandemie sind hingegen Einschränkungen des gesellschaftlichen Lebens, vor allem zum Schutz besonders gefährdeter Patientengruppen und zur Aufrechterhaltung eines funktionierenden Gesundheitssystems. In Übereinstimmung mit RKI (Robert Koch-Institut) und WHO (Weltgesundheitsorganisation) empfehlen wir in der aktuellen Pandemie-Situation Präventionsmaßnahmen, wie beispielsweise [22–25]:

- Abstand von mindestens 1,5–2 m zu anderen Personen halten,
- Einhaltung von allgemeinen Hygienemaßnahmen, wie regelmäßige Händedesinfektion/regelmäßiges Händewaschen für mindestens 30 s, Berührung von Schleimhäuten mit den Händen vermeiden,
- Minimierung der sozialen Kontakte,
- Beschränkung von persönlichen Patientenkontakten auf das absolut Notwendige,
- Tragen von persönlicher Schutzkleidung und
- regelmäßige Flächendesinfektion, insbesondere Türklinken etc.,
- möglichst rasche und umfassende Impfkampagnen.
